# Detection of Carbapenem-Resistant Genes in *Escherichia coli* Isolated from Drinking Water in Khartoum, Sudan

**DOI:** 10.1155/2020/2571293

**Published:** 2020-06-12

**Authors:** Neama Esmat Mahmoud, Hisham N. Altayb, Reem Majzoub Gurashi

**Affiliations:** ^1^Department of Microbiology, Faculty of Medical Laboratories Sciences, Alneelain University, Khartoum, Sudan; ^2^Biochemistry Department, Faculty of Sciences, King Abdulaziz University, Jeddah 21452, Saudi Arabia

## Abstract

Waterborne *Escherichia coli* are a major reservoir of antimicrobial resistance (AMR). Carbapenem-resistance, especially when mediated by transferable carbapenemase-encoding genes, is spreading worldwide and causing dramatically limiting treatment options. In our country, studies for the detection of carbapenem resistance in drinking water do not exist; therefore, this work was carried out to determine the prevalence of carbapenem-resistant genes “bla_KPC_, bla_IMP_, bla_NDM_, bla_SPM_, bla_VIM_, and bla_OXA-48_” among *Escherichia coli* isolated from drinking water in Khartoum, Sudan. A total of forty-five *E. coli* bacteria were isolated from different sources of drinking water. Antimicrobial susceptibility testing was performed using imipenem (10 mg/disc), gentamicin (10 mg/disc), ceftriaxone (30 mg/disc), ciprofloxacin (5 mg/disc), chloramphenicol (30 mg/disc), and tetracycline (30 mg/disc). “Sensitive” or “resistant” patterns of *E. coli* were judged using antibiotic minimum inhibitory concentration (MIC). Bacterial genomic DNA was extracted by the boiling method, and then multiplex polymerase chain reaction was performed to detect the carbapenemase genes (bla_KPC_, bla_IMP_, *bla*_*NDM*_, bla_SPM_, bla_VIM_, and bla_OXA-48_). Multiplex PCR assays confirmed the presence of carbapenemase genes in 28% of all water isolates. OXA-48 gene was the most predominant gene, detected in 15.5% of the isolates. The bla_KPC_ and *bla*_*SPM*_ genes were also detected in 4.4% and 8.8% of the isolates, respectively. However, the isolates were negative for *bla*_*NDM*_, bla_VIM_, and bla_IMP_ genes. The isolates showed a high rate of tetracycline resistance (97.7%), followed by gentamicin (57.7%), ciprofloxacin (46.6%), ceftriaxone (35.5%), and chloramphenicol (31.1%). In conclusion, this study confirmed for the first time the presence of *E. coli* carried carbapenem-resistant genes in the drinking water of Khartoum state, Sudan. These isolates commonly carried OXA-48 (7/45), followed by SPM (4/45) and KPC (2/45).

## 1. Introduction

Good health depends on clean, safe water supply. However, it should be free from toxic chemical and pathogenic microorganisms [1]. The determination that water is consequently unsafe to drink is by focusing on detecting fecal contamination. High fecal levels can mean that water contains pathogens [2]. *Escherichia coli* are often used as an indicator of fecal contamination of drinking water, and ingestion of water contaminated with *E. coli* can lead to serious complications including diarrhea, enteritis, and mortality in some cases [3–5]. Antimicrobial-resistant (AMR) bacteria are widespread in environmental waters, and *E. coli* is a concerning reservoir for AMR due to its plenty in such environments with a high risk of transfer from the environment to humans [6–8].

*Escherichia coli* have become intrinsically resistant to many types of *β*-lactamase due to the emergence of organisms carrying extended-spectrum b-lactamases (ESBLs) and plasmid-mediated AmpC *β*-lactamases [9]. Carbapenems, among the beta-lactams, are the most effective agents for the treatment of severe infection caused by ESBL-positive *E. coli*, and their unique molecular structure is due to the presence of a carbapenem together with the beta-lactam ring [10–12]. Resistance to carbapenems occurs due to the production of carbapenemases, a class of enzymes capable of hydrolyzing carbapenems and other *β*-lactams; the other mechanisms can be by the poor binding of carbapenems to penicillin-binding proteins present in the bacteria, lack of porins present in the bacterial cell membrane, and overexpression of multidrug efflux pumps by the bacteria [9, 13]. They are classes of enzymes capable of hydrolyzing carbapenems and other *β*-lactams, including classes A, B, and D. KPCs are the most frequently encountered enzymes in class A carbapenemases. Class B metallo-*β*-lactamases (MBLs) are mostly of the VIM, IMP, SPM, GM, SIM, and NDM. Class D *β*-lactamases, also named OXAs, OXA-48, represent the main enzyme isolated around the world [14].

*Klebsiella pneumoniae* carbapenemase (KPC) was first identified in the USA and then continue to spread globally [14]. New Delhi metallo-beta-lactamases (NDM-1) is rapidly spread since it is detected in India and disseminated through patients traveled to many countries [14]. Imipenemase (IMP) and Verona imipenemase (VIM) type metallo-beta-lactamases (MBLs) were first identified in Japan and Italy, respectively, and have successfully spread to all continents since then. Sao Paolo metallo-*β*-lactamase (SPM) is more geographically restricted and remains mostly confined in South America. Oxacillinase (OXA-48) is the most prevalent in the Middle East and North Africa, as well as other countries worldwide [10, 11, 15].

The quality of drinking water has an important role in human infection and disease. Contaminated water plays an important role in the transmission of carbapenem-resistant *E. coli* to humans. The dissemination of the resistance gene represents a serious public health threat, which causes dangerous limitation of treatment option, and significant mortality rate [1, 2]. Our study aimed at determining the prevalence of carbapenem-resistant genes “bla_KPC_, bla_IMP_, bla_NDM_, bla_SPM_, bla_VIM_, and bla_OXA-48_” among *Escherichia coli* isolated from drinking water in Khartoum, Sudan.

## 2. Materials and Methods

### 2.1. Sample Collection and Isolation of *E. coli*

Forty-five isolates of *E. coli* were identified from 150 water samples in the central public health laboratory in Khartoum, Sudan, from January to June 2017. Water samples were collected from different water sources, including taps (40), cooler (40), and tanks (70) in houses. The samples were cultured in two bottles of Brilliant Green Bile Broth (BGBB) media with Durham tube and incubated for 48 hours in 37°C; after incubation, turbidity and gas represented the presence of coliform, the positive samples were purified in EMB media and MacConkey agar, and then they were identified using Gram stain and biochemical tests including Kliger iron agar, indole, citrate, and urease tests [16, 17].

### 2.2. Antibiotic Susceptibility Test

Forty-five isolates were screened for antimicrobial resistance using the Kirby-Bauer disc diffusion method. Six antibiotics tested included imipenem (10 mg/disc), gentamicin (10 mg/disc), ceftriaxone (30 mg/disc), ciprofloxacin (5 mg/disc), chloramphenicol (30 mg/disc), and tetracycline (30 mg/disc). “Sensitive,” “intermediate resistant,” or “resistant” patterns of *E. coli* were judged using antibiotic minimum inhibitory concentration (MIC) provided by the guidelines of the Clinical and Laboratory Standards Institute (CLSI). *E. coli* strain ATCC, no. 25922, was used as quality control. All isolates showing “resistant” or “intermediate resistant” patterns were classified as “resistant” [6, 18].

### 2.3. DNA Extraction

Bacterial DNA was extracted using the boiling method. Firstly, a single colony from all bacterial strains was subcultured on Muller-Hinton agar, and after overnight incubation, several colonies were emulsified in a test tube containing 1 ml of the distilled water and boiled at 95°C for 10 minutes, and then the supernatant containing DNA was transferred to a new Eppendorf tube [19].

### 2.4. Multiplex PCR for the Detection of Carbapenemase Genes

The presence of the carbapenemases genes was determined using gene-specific primer targeting *bla*_*NDM*_*, bla*_*IMP*_*, bla*_*VIM,*_*bla*_*SPM*_*, bla*_*KPC*_, and *bla*_*OXA_48*._ The primers were obtained from the Macrogen Company, Korea (Table 1).

#### 2.4.1. Method for Detection of *bla*_*NDM*_, *bla*_*IMP*_, *bla*_*VIM*_, *bla*_*SPM*_, *bla*_*KPC*_, *and bla*_*OXA_48*_

PCR was carried out in a total reaction volume of 20 *µ*l consisting of 0.6 *µ* forward and 0.6 *µ* of the reverse primer for each gene, and 5 *µ*l of extracted DNA and 8.8 *µ*l of distilled water were added to 5 *µ*l of PCR master mix (Maximum PCR Premix Kit i-Taq, iNtRON, Biotechnology, Korea). For *bla*_*KPC*_ and *bla*_*OXA_48*_, the same volume from primers and extracted DNA was added to 8.2 of distilled water and 5 *µ*l PCR master mix to reach the final volume of 20 *µ*l. *E. coli* harbored KPC gene was used as a positive control [20].

#### 2.4.2. PCR Amplification

The thermal cycling machine (TECHNE 412, UK) was programmed for 35 cycles for DNA amplification. The program consists of an initial denaturation step at 95°C for 5 minutes, denaturation and annealing at 94°C and 60°C, respectively, for 45 seconds, and elongation for one minute at 72°C. The same program was used for *bla*_*KPC*_, and *bla*_*OXA_48*_, except annealing temperature, was adjusted to 55°C for 45 seconds, and all primer sets had a final extension of 72°C for 7 minutes [20, 21].

#### 2.4.3. Visualization of Genome

Gel electrophoresis was done after PCR to visualize bands. 1.5 *µ*l from ethidium bromide was added to 2% agarose gel and poured into the casting tray for solidification, and then 5 *µ*l from extracted DNA was placed in the wells. The voltage applied was 60 volts, and the current was adjusted to 100 Amperes for 30 minutes. After that, the PCR product was visualized using a UV transilluminator [22].

### 2.5. Data Analysis

The collected data were analyzed by the statistical package of social science (SPSS) program version 21.0; the chi-square test was used to assess the association between variables, and the *p* value that considered significant was less than 0.05 [23].

## 3. Results

**Figure 1:**
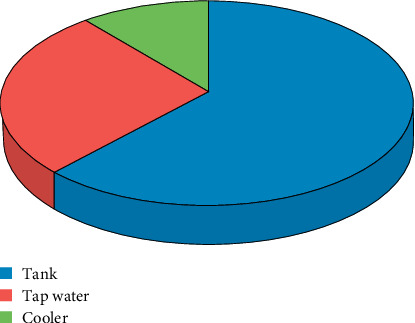
The prevalence of *E. coli* in different drinking water sources.

Forty-five isolates were successfully grown on BGBB media and confirmed as *E*. *coli* by the biochemical test. Twenty-eight isolates were obtained from tanks, whereas 12 and 5 isolates were collected from tap water and cooler, respectively. The result showed that there was a high frequency of *E. coli* isolates in tanks (28/70, 40%) and low frequency of isolates in tap water and cooler (12/40, 30%, and 5/40, 12.5%), respectively. The prevalence of *E. coli* in drinking water source is shown in Figure 1.

### 3.1. Susceptibility Testing

Carbapenem: the antimicrobial susceptibility pattern showed 33% (15/45) of isolates were resistant to imipenem, while 66% (30/45) were susceptible.

Other antibiotics: resistance to tetracycline (97.7%) was the highest among the antibiotics used, followed by gentamicin (57.7%), ciprofloxacin (46.6%), ceftriaxone (35.5%), and chloramphenicol (31.1%).

### 3.2. Detection of Carbapenem-Resistant Genes

**Figure 2:**
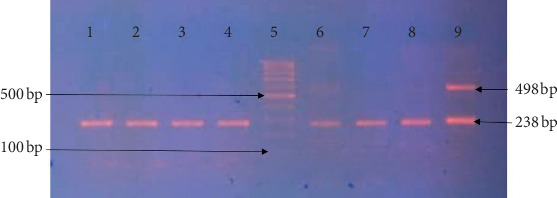
Multiplex PCR for amplification of *bla*_*OXA-48*_ and *bla*_*KPC*_ genes on 2% agarose gel electrophoresis: lane 5: DNA ladder 100 bp; lanes 1, 2, 3, 4, 6, 7, 8, and 9 are OXA-48 positive samples showing a typical band size of (238 bp). Lane 9 is a positive sample for *bla*_*KPC*_ showing a typical band size of (498 bp).

**Figure 3:**
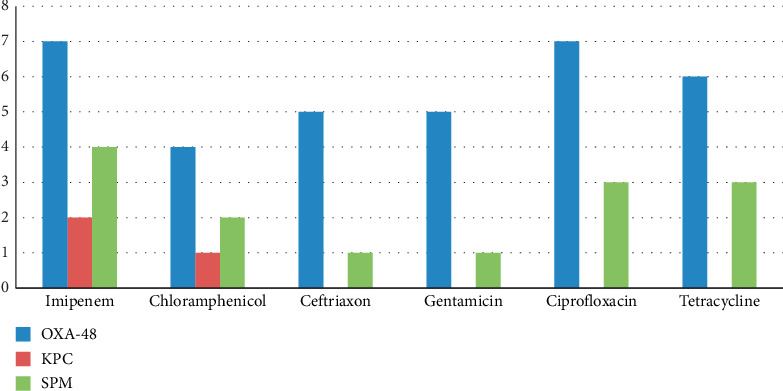
Antibiotics resistance rate among *E. coli* harbored carbapenem-resistant genes.

Based on PCR assay, twenty-eight percent (13/45) of the isolates carried one carbapenem gene. Predominantly 7 isolates (15.5%) were positive for *bla*_*OXA-48*_, followed by *bla*_*SPM*_ (8.8%, 4/45) and *bla*_*KPC*_ (4.4%, 2/45), while all isolates lack the other three genes *bla*_*NDM*_, *bla*_*VIM*_, and *bla*_*IMP*_. PCR amplification for genes product is shown in Figure 2. The number of bacterial strains positive for carbapenem-resistant genes is illustrated in Table 2, while Table 3 and Figure 3 demonstrate the association between antibiotic resistance and the presence of carbapenem-resistant genes.

## 4. Discussion

*Escherichia coli* is one of the major etiologic agents for urinary tract infection, sepsis, enteritis, and meningitis; in addition, *E. coli* has a common role in contaminated drinking water that can lead to a serious complication including diarrhea and enteritis [4, 12]. Carbapenem-resistant *E. coli* is an important public health concern worldwide. The presence of this resistant bacteria may contribute to increasing the risk to human health. So it is important to have more information regarding such issues [16, 24, 25]. In the present study, we found that 13/15 (86%) of imipenem resistance isolates were positive for *bla*_*OXA*_, *bla*_*SPM*_, and *bla*_*KPC*_ genes. 15.5% of isolates (7/45) harbored the *bla*_*OXA-48*_ gene, the most predominant gene. There was a significant association (*p* value = 0.003) between the presence of the *bla*_*OXA-48*_ gene and water sources (tap water, cooler, and tanks). This finding is in agreement with a study conducted by Amaya et al. in Nicaragua; they reported that gene encoding for *bla*_*OXA*_ was detected in 1% of well-water samples [26]. There was another study done by Tanner et al.; they identified *bla*_*OXA-48*_-type genes (3.9%) in US drinking water. In contrast, the same study was not in agreement with our result. However, the *bla*_*KPC*_ gene was not detected. This variation may be due to differences in the collection time and area [27]. *bla*_*KPC*_ gene was found in 4.4% of total isolates (2/45). This is in agreement with the result obtained by Haberecht et al. in Northern Colorado in which *E. coli* isolated from environmental water represented 4.2% of total suspected *bla*_*KPC*_ [6]. Although studies reported the presence of *bla*_*SPM*_ gene in *E. coli* isolated from water was limited, our result showed the prevalence of this gene is 8.8% in drinking water. But there was no significant correlation between the water source and *bla*_*SPM*_ gene.

On the other hand, the isolates were negative for *bla*_*NDM*_, *bla*_*VIM*_, *and bla*_*IMP*_ genes. The result is comparable to the study done by Petron et al. in Morocco, which found a similar finding among *Serratia marcescens* isolated from water specimens [28]. But this finding is in disagreement with a study conducted in the USA by Hoelle et al., which showed that about 55% of *E. coli* isolates were positive for VIM gene and 1% were positive for IMP gene [29]. These variations in the results may be explained by the differences in the time of study and their large sample size.

In the current study, we detected the carbapenemase gene in two bacterial species with phenotypical resistance to carbapenem drugs, but its resistance mechanisms were not detected by any of the screened carbapenemase primers used in this study. This might be due to the limited number of genes targeted in our study as well as to other mechanisms of resistance, such as porin loss/mutations [30].

Limitations of the present study include that a few samples were used and that did not represent a large population number. Also, the current research did not use primers to target all known carbapenemases genes. Despite these limitations, the study has provided the distribution of the common carbapenemase genes and the magnitude of the problem. Although the study did not investigate the clonality of the isolates and the sequence of the genes, tests based on molecular techniques are considered the standard tests for the identification of carbapenemase genes and multiplex-PCR as reliable, fast method for the detection of these genes [11].

## 5. Conclusion

There is a high frequency of *E. coli* harbored carbapenemase gene (*bla*_*OXA-48*_, *bla*_*SPM*_, and *bla*_*KPC*_) in drinking water in Khartoum State, Sudan. We found twenty-eight of *E. coli* isolates carried carbapenemase genes and tested as resistant to imipenem (13/45). *bla*_*OXA-48*_ (15.5) gene is the most predominant gene among positive isolates, followed by *bla*_*SPM*_ (8.8) and *bla*_*KPC*_ (4.4) genes. Resistance to tetracycline and chloramphenicol is also high, and it may be due to that the plasmid carries carbapenem-resistant genes, and it may carry other resistant genes. Also, it was found that there is no association (*p* value>0.05) with gentamicin, ceftriaxone, and ciprofloxacin antibiotics and the presence of carbapenem-resistant genes.

Further study using a large sample size is required to determine the prevalence of carbapenemase genes in drinking water. Although the investigation of clonality for the isolates and sequencing of the genes is important to be adequately characterized, the PCR technique provides a satisfied and reliable result for the detection of carbapenemase genes.

## Figures and Tables

**Table 1:** Primer sequences used in this study.

Primer name	Sequence (5′-3′)	Amplicon size (Pb)	References
*bla*_*SPM*_	Forward: AAAATCTGGGTACGCAAACG Reverse: ACATTATCCGCTGGAACA	271	[20]
*bla*_*VIM*_	Forward: GGTGTTTGGTCGCATATCGCAA Reverse: ATTCAGCCAGATCGGCATCGGC	502	[20]
*bla*_*IPM*_	Forward: TCGTTTGAAGAAGTTAACG Reverse: ATGTAAGTTTCAAGAGTGATGC	568	[20]
*bla*_*NDM*_	Forward: GGTTTGGCGATCTGGTTTTC Reverse: CGGAATGGCTCATCACGATC	624	[20]
*bla*_*KPC*_	Forward: CATTCAAGGGCTTTCTTGCTGC Reverse: ACGACGGCATAGTCATTTGC	498	[21]
*bla*_*OXA-48*_	Forward: GCTTGATCGCCCTCGATT Reverse: GATTTGCTCCGTGGCCGAAA	238	[21]

**Table 2:** The number of bacterial strains positive for carbapenem-resistant genes.

Genes	Number of bacteria strains
*bla*_*OXA-48*_	7
*bla*_*KPC*_	2
*bla*_*SPM*_	4

**Table 3:** The association between antibiotic resistance and the presence of carbapenem-resistant genes.

Genes	Imipenem	Chloramphenicol	Ceftriaxone	Gentamicin	Ciprofloxacin	Tetracycline
*bla*_*OXA-48*_	7	4	5	5	7	6
*bla*_*KPC*_	2	1	0	1	1	2
*bla*_*SPM*_	4	2	1	1	3	2

## Data Availability

The data that support the descriptive statistical analyses of sample characteristics and resistance gene presence are available from the corresponding author upon reasonable request.
